# Mathematical model of the anatomy and fibre orientation field of the left ventricle of the heart

**DOI:** 10.1186/1475-925X-12-54

**Published:** 2013-06-18

**Authors:** Sergey F Pravdin, Vitaly I Berdyshev, Alexander V Panfilov, Leonid B Katsnelson, Olga Solovyova, Vladimir S Markhasin

**Affiliations:** 1 Function Approximation Theory Department, Institute of Mathematics and Mechanics, Ekaterinburg, Russia; 2 Laboratory of Mathematical Physiology, Institute of Immunology and Physiology, Ekaterinburg, Russia; 3 Department of Physics and Astronomy, Ghent University, Krijgslaan 281, S9, Ghent, 9000, Belgium; 4, Ural Federal University, Ekaterinburg, Russia

**Keywords:** Mathematical anatomy, Left ventricle of the mammalian heart, Mathematical modelling of the cardiac form and structure

## Abstract

**Background:**

One of the main factors affecting propagation of electrical waves and contraction in ventricles of the heart is anisotropy of cardiac tissue. Anisotropy is determined by orientation of myocardial fibres. Determining fibre orientation field and shape of the heart is important for anatomically accurate modelling of electrical and mechanical function of the heart. The aim of this paper is to introduce a theoretical rule-based model for anatomy and fibre orientation of the left ventricle (LV) of the heart and to compare it with experimental data. We suggest explicit analytical formulae that allow us to obtain the left ventricle form and its fibre direction field. The ventricle band concept of cardiac architecture given by Torrent-Guasp is chosen as the model postulate.

**Methods:**

In our approach, anisotropy of the heart is derived from some general principles. The LV is considered as a set of identical spiral surfaces, each of which can be produced from the other by rotation around one vertical axis. Each spiral surface is filled with non-intersecting curves which represent myocardial fibres.

For model verification, we use experimental data on fibre orientation in human and canine hearts.

**Results:**

LV shape and anisotropy are represented by explicit analytical expressions in a curvilinear 3-D coordinate system. The derived fibre orientation field shows good qualitative agreement with experimental data. The model reveals the most thorough quantitative simulation of fibre angles at the LV middle zone.

**Conclusions:**

Our analysis shows that the band concept can generate realistic anisotropy of the LV. Our model shows good qualitative agreement between the simulated fibre orientation field and the experimental data on LV anisotropy, and the model can be used for various numerical simulations to study the effects of anisotropy on cardiac excitation and mechanical function.

## Background

Modern models of complex physiological systems, such as the heart, integrate description from the molecular to the whole organ level and allow researchers to study mechanisms of both mechanical and electrical cardiac activity in normal and pathological hearts.

Over the last several years, a number of models describing electrical and/or mechanical function of the whole heart or its chambers have been proposed [1-12]. The most recent of them consider detailed description of cardiac anatomy and fibre orientation fields as crucial factors for correct representation of the physiological features that are central to heart function.

All approaches to representing cardiac anatomy and anisotropy can be subdivided roughly into two large groups: the individual map approaches, in which fibre orientation is directly measured in the heart using various experimental techniques; and theoretical approaches, in which fibre orientation is generated by algorithms.

In this article, we suggest a theoretical model for anatomy and fibre orientation of the LV. The model is based on the ventricle band concept of cardiac architecture given by Torrent-Guasp [13]. In 1972, Torrent-Guasp proposed an anatomic concept in which both right and left heart ventricles were considered segments of a single myofibre band twisted and wrapped into a double helical coil [13]. Since that time, this concept has been a subject of intense discussion. Many cardiac anatomists [14,15] consider the Torrent-Guasp hypothesis a gross simplification, and a number of imaging scientists propose a more complex organization of the LV micro-architecture [16]. Another group of researchers has a favorable view on the ventricle band concept [17-19]. For example, an article signed by more than 20 prominent scientists [20] concludes that ‘models such as that of Torrent-Guasp et al., which proposes conduction along fibre orientation in a single muscular band and defies conventional concepts of activation, should be investigated’. In spite of that interest, the Torrent-Guasp model was never formalized and compared to data on measured cardiac anatomy. Note that in our view, features of the model, such as the possibility of representing realistic fibre orientations by a single warped band, can be proved or disproved only by means of mathematical modelling. Regardless of the outcome, such a formulation will be useful.

In this article, we follow Torrent-Guasp’s approach to build cardiac anatomical models of increasing complexity that also use later measurements by Streeter [21]. We show that this description allows one to represent such properties of heart anisotropy as fibre rotation and its dependence on the latitude, spiralling of fibres at the apex and fibre’s maximal angle of torsion about the LV axis. We also perform quantitative comparisons with data from Streeter [21] and Hunter [22], showing good correspondence of the measured fibre orientation fields with that given by our model.

In our model, both the anatomy and fibre orientation field are precisely formulated mathematically. This allows a researcher to apply analytical methods to investigate cardiac electrophysiology and mechanics. In addition, any variations in the shape of the LV and the anisotropy pattern can be reproduced easily by this approach.

## The construction of the LV model

The description of the model consists of several steps, starting with simple shapes and approaching the final LV model. Initial steps follow the representation of Pettigrew’s idea (one can see a copy of his figure in Streeter’s work [21] (see Figure three)). We then modify its description and obtain the LV model.

### A semicircle with chords on it

Pettigrew began his construction from a semicircle with a set of curves on it [23], as one can see in Streeter’s paper ([21], Figure three a). We describe the figure analytically as follows.

Consider a semicircle with radius *K* given in the polar coordinate system (P,*Φ*):

(1) 0≤P≤K,0≤Φ≤π.

**Figure 1:**
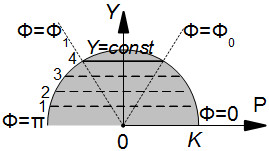
**Horizontal chords on the semicircle.** The example chords are numbered so that they can be identified after transformations that will be shown below.

Following Pettigrew [23], let us construct a sequence of horizontal chords (Figure 1):

(2) Y=const,0<Y<K,

(3) $$\Phi_{0}:=\text{arcsin}\frac{Y}{K}\leq \Phi \leq \pi -\text{arcsin}\frac{Y}{K}=:\Phi_{1},$$

(4) $$P(\Phi )=\frac{Y}{\text{sin}\Phi }.$$

Here, *Φ*_0_ and *Φ*_1_ are polar angles of the right and the left ends of a chord.

A global idea of the model is to wrap this semicircle onto a surface (e.g. conical). The curves will give fibre orientation on that surface, and then rotation of such a surface will give a 3-D structure of the heart. The first surface that we will construct is a simple cone. We use a semicircle and a cone because the possibility to wrap a sector of circle to a simple cone is a proven mathematical fact and the LV form closely resembles a cone. We consider the semicircle as the part of the Torrent-Guasp “unique myocardial band” which corresponds to the LV. After this wrapping, we will convert the cone to a more complex surface by a non-linear transformation which will allow us to obtain a more feasible LV model.

### Wrapping the semicircle to a cone

In ([21] Figure three (b-c)), one can see a wrapping of the semicircle to a surface. We propose the following analytical description of the procedure.

**Figure 2:**
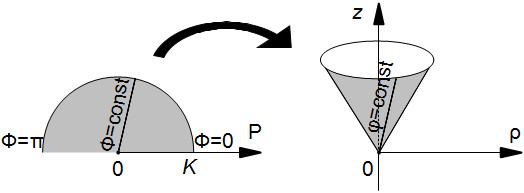
Wrapping of the semicircle to a conic surface.

Let us imagine that the semicircle is made of paper. We can wrap this semicircle to a right circular cone (maybe partial or with an overlap) so that the cone vertex corresponds to the semicircle centre (Figure 2). Let us denote an angle along the cone arc as *cone twist angleϕ*_max_. We get a cone that becomes closed, if *ϕ*_max_ = 2 *π*. We are going to consider only the case where *ϕ*_max_ > *π*. The cone is specified in a cylindrical coordinate system (*ρ*, *ϕ*, *z*) as follows.

First note that curves described by *Φ* = const do not bend during wrapping. Therefore, these curves *Φ* = const are the generatrices of the cone. The parametric equations of the cone are

(5) $$\rho (P,\Phi )=P\cdot \frac{\pi }{\phi_{\text{max}}},$$

(6) $$\phi (P,\Phi )=\Phi \cdot \frac{\phi_{\text{max}}}{\pi },$$

(7) $$z(P,\Phi )=P\sqrt{1-{\left(\frac{\pi }{\phi_{\text{max}}}\right)}^{2}}.$$

An explicit equation for the object is

(8) $$z(\rho ,\phi )=\rho \sqrt{{\left(\frac{\phi_{\text{max}}}{\pi }\right)}^{2}-1}.$$

Let us note that the semicircumference, limiting the given semicircle, transforms to the cone planar arc, and the centre of the circle becomes the cone’s apex.

In order to model not only muscular layers, but also myofibres, one has to look for the position of the transformed chords after the wrapping.

Let us find the chord *Y* = const images on the conical surface, by substituting (4) into (5), (6), (7):

(9) $$\rho (\Phi )=\frac{Y}{\text{sin}\Phi }\cdot \frac{\pi }{\phi_{\text{max}}},$$

(10) $$\phi (\Phi )=\Phi \cdot \frac{\phi_{\text{max}}}{\pi },$$

(11) $$z(\Phi )=\frac{Y}{\text{sin}\Phi }\cdot \sqrt{{\left(\frac{\phi_{\text{max}}}{\pi }\right)}^{2}-1}.$$

**Figure 3:**
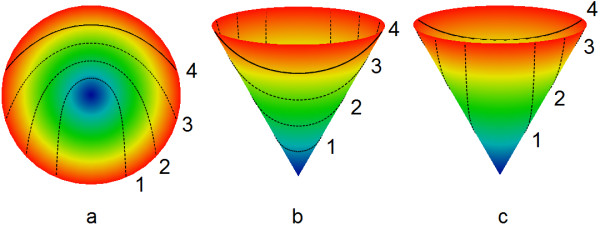
**A conical surface with wrapping angle of 2π and a series of chord images.** Bottom view (**a**); viewing from the top at an oblique angle from two different directions (**b** and **c**). Colour represents height, and the chord images are drawn in black. The numbering and line styles of the chord images and of the chords here and in Figure 1 are the same.

The results can be seen in Figure 3. It is not difficult to see that rotation of such a simple conical surface around the vertical axis does not give a good representation of the heart anatomy, as it will produce only a conical surface, i.e. a body of zero thickness. To improve that, we will generalize the procedure by introducing dependency of the generatrices on the rotation angle.

### Construction of spiral surfaces

In Figure 2, the cone’s generatrix was *z* = *kρ*, where *k* was a constant (see (8)), so that it did not depend on the angle *ϕ*. Let us consider the more general situation when *z* = _*ϕ*_(*ρ*). Such a generatrix will generate a spiral surface, which will finally give us a proper representation of the heart’s geometry.

Let us consider a few examples.

**Figure 4:**
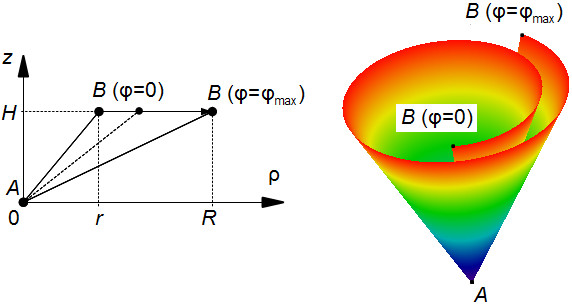
**On the left: The construction of the generatrices for a spiral surface, corresponding to the conical LV with a dot vertex.** The graph of the function _*ϕ*_(*ρ*) (see (12)) connects the points *A* and *B* for *ϕ* = 0 and *ϕ* = *ϕ*_max_ by the line segments. On the right: The resulting pseudoconus.

We can first assume that generatrix changes its slope as shown in Figure 4. It can be formally represented as

(12) $$Z_{\phi }(\rho )=\frac{H}{r+\gamma (R-r)}\cdot \rho ,$$

where *H*,*R*,*r* are positive constants, and

(13) $$\gamma =\frac{\phi }{\phi_{\text{max}}},$$

with 0 ≤ *γ* ≤ 1. The domain of _*ϕ*_ is taken

$$D(Z_{\phi })=\left[0,r+\gamma (R-r)\right],$$

such that the codomain is

$$E(Z_{\phi })=\;[0,H].$$

The conical shape produced by such generatrices is shown in Figure 4, on the right. We will call it *a pseudoconical surface*.

If we rotate a pseudoconical surface around the vertical axis, we get a conical body that has some resemblance to the LV (it has an LV cavity), but its thickness at the apex will be zero. To improve, we modify the generatrices as follows.

**Figure 5:**
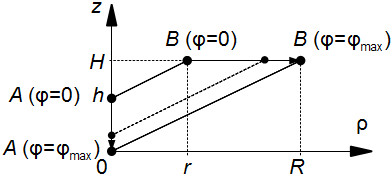
**The construction of the generating lines for a spiral surface corresponding to the conical LV with a thick vertex.** The graph of the function _*ϕ*_(*ρ*) (see (14)) connects the points *A* and *B* for *ϕ* = 0 and *ϕ* = *ϕ*_max_ by the line segments.

Let us move the end *A* (see Figure 4) down by a value *hγ*, i.e. proportional to the angle *ϕ* = *γϕ*_max_ (*h* is a positive constant), as shown in Figure 5:

(14) $$Z_{\phi }(\rho )=\frac{H-h+\mathrm{h\gamma}}{r+\gamma (R-r)}\cdot \rho +h-\mathrm{h\gamma},$$

$$D(Z_{\phi })=\left[0,r+\gamma (R-r)\right],\;E(Z_{\phi })=\left[h-\mathrm{h\gamma},H\right].$$

As a result, the thickness of the LV at the apex will become *h* > 0, which improves our representation. However, a real LV surface is not conical, so we have to use curves as generatrices instead of straight line segments.

**Figure 6:**
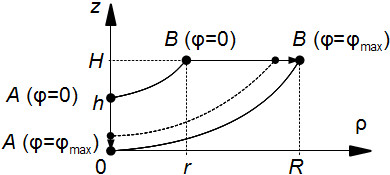
**The construction of the generatrices for the salient spiral surface used in modelling the convex LV with a thick vertex.** The graph of the function _*ϕ*_(*ρ*) (see (15)) connects the points *A* and *B* for *ϕ* = 0 and *ϕ* = *ϕ*_max_ by the arcs of curves.

Let us connect the same points *A* and *B*, as in the previous example, but by an arc (Figure 6):

(15) $$Z_{\phi }(\rho )=\left(H-h+\mathrm{h\gamma}\right)\cdot F_{\phi }\left(\frac{\rho }{r+\gamma (R-r)}\right)+h-\mathrm{h\gamma},$$

where the function _*ϕ*_ represents a curved generatrix.

$$D(F_{\phi })=E(F_{\phi })=\;[0,1];$$

$$D(Z_{\phi })=\left[0,r+\gamma (R-r)\right],\;E(Z_{\phi })=\left[h-\mathrm{h\gamma},H\right].$$

The following properties are imposed on the function _*ϕ*_(*ρ*) 

1. *D*(_*ϕ*_(*ρ*)) = *E*(_*ϕ*_(*ρ*)) = [0,1]

2. _*ϕ*_(*ρ*) is continuous and differentiable on [0,1]

3. _*ϕ*_(*ρ*) increases monotonically on [0,1].

We refer to the functions with these properties as *generating functions* (GF). In the first example $F_{\phi }(t)=t,\;Z_{\phi }(\rho )=F_{\phi }\left(\frac{\rho }{r+\gamma (R-r)}\right)\cdot H;$ in the second example _*ϕ*_(*t*) = *t*, and is represented through in the same way as in the third example (see (15)). Now let us choose a proper GF to construct a more realistic LV.

### A model of the LV

To represent the shape of the epicardial LV surface, Streeter ([21], pp. 91 – 92) used the following functions:

(16) $$\rho_{\text{epi}}(\psi )=R_{b}\left(\epsilon \text{cos}\psi +(1-\epsilon )(1-\text{sin}\psi )\right);$$

(17) $$z_{\text{epi}}(\psi )=Z_{b}(1-\text{sin}\psi ).$$

**Figure 7:**
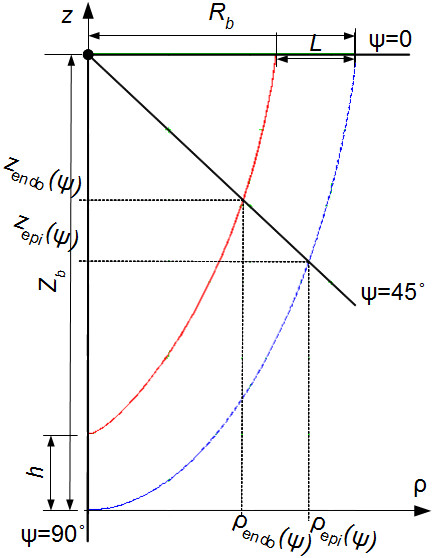
**A shape of function representing the endocardial (the solid red line, see **(16), (17)) **and epicardial (the dashed blue line, see **(**18**), (**19**)) surfaces.

If *ϵ* = 0, the curve *AB* is a line segment, and if *ϵ* = 1, it is a quarter of an ellipse. For intermediate *ϵ* values, we get intermediate curves. Let us use the following equation of the endocardium in analogy with Streeter’s description:

(18) $$\rho_{\text{endo}}(\psi )=(R_{b}-L)\left(\epsilon \text{cos}\psi +(1-\epsilon )(1-\text{sin}\psi )\right);$$

(19) $$z_{\text{endo}}(\psi )=(Z_{b}-h)(1-\text{sin}\psi )+h,$$

where “latitude” *ψ* takes values 0 ° ≤ *ψ* ≤ 90° (Figure 7). Parameters determining the shape are: an outer radius *R*_*b*_ near the equator; a thickness *L* near the equator; a height *Z*_*b*_; a thickness *h* at the apex.

The form of any intermediate layer between epi- and endocardium is described by the equations (see also Additional file 1):

$$\rho_{\text{mid}}(\psi ,\gamma )=(R_{b}-(1-\gamma )L)(\epsilon \text{cos}\psi +(1-\epsilon )(1-\text{sin}\psi )),$$

$$z_{\text{mid}}(\psi ,\gamma )=(Z_{b}-(1-\gamma )h)(1-\text{sin}\psi )+(1-\gamma )\mathrm{h.}$$

Elimination of *ψ* delivers an explicit equation for the surface:

(20) $$z_{\text{epi}}(\rho )=Z_{b}F_{\epsilon \text{sp}}(\rho /R_{b});$$

(21) $$z_{\text{endo}}(\rho )=(Z_{b}-h)F_{\epsilon \text{sp}}(\rho /(R_{b}-L))+h,$$

(22) $$F_{\epsilon \text{sp}}(t)=\frac{\epsilon^{2}+t(1-\epsilon )-\epsilon \sqrt{2t(1-\epsilon )+\epsilon^{2}-t^{2}}}{{\left(1-\epsilon \right)}^{2}+\epsilon^{2}},$$

where _*ϵ*sp_: [0,1] → [0,1] is the GF of the epicardium, the endocardium and every intermediate layer of the LV wall; *ρ*_epi_/*R*_*b*_ ∈ [0,1], *ρ*_endo_/(*R*_*b*_ - *L*) ∈ [0,1].

As a result, we get the following definition of the *ϵ* - spiral surface (ESS, see Additional files 2 and 3):

(23) $$z_{\epsilon \text{sp}}(\rho ,\phi )=(Z_{b}-(1-\gamma )h)F_{\epsilon \text{sp}}\left(\frac{\rho }{R_{b}-(1-\gamma )L}\right)+(1-\gamma )\mathrm{h.}$$

**Figure 8:**
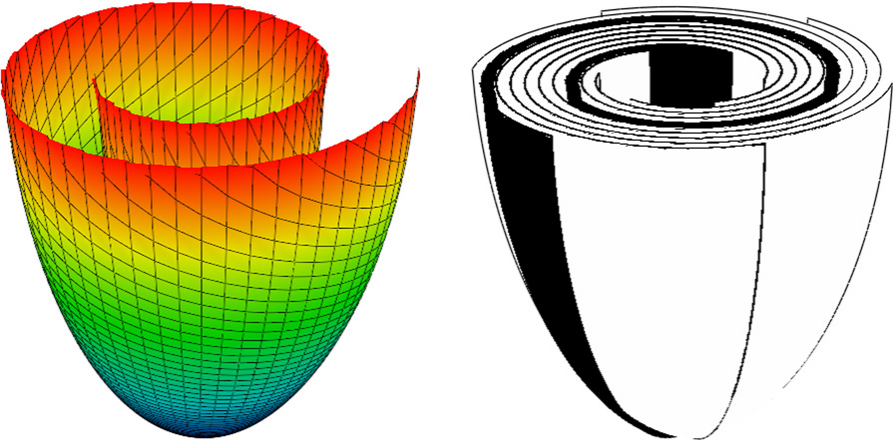
**A spiral surface (on the left, the lines have equations *ρ * = const and *Φ * = const), a schematic representation of the LV obtained (six spiral surfaces rotated by angles 0°, 60°, 120°, 180°, 240° and 300° around the vertical axis (on the right, compare with ([**[21]**], Figure three D))).** If we consider infinitely many such surfaces, we obtain the whole LV model.

The spiral surface’s border with an angle *ϕ* = 0 is located at the endocardial side, and the border with an angle *ϕ* = *ϕ*_max_ lies at the epicardial side. The LV model is made as a body of revolution of an ESS. See Figure 8 for an example of a single and multiple nested ESS. We form the LV by using shifted layers (or the rotated spiral surfaces, which is the same) because a thick muscular layer cannot be wrapped to a body of revolution with overlapping, but without any shift of its sheets.

Our description reproduces the form of epicardium from Streeter’s work (see Figure forty in [21]), but for endocardium, Streeter uses more complex equations with an additional parameter that he called the ‘angle of taper’. However, we found that even without this parameter our function reasonably reproduces the form of the endocardium, and, thus, we decided to use equation (23) without further modifications.

**Figure 9:**
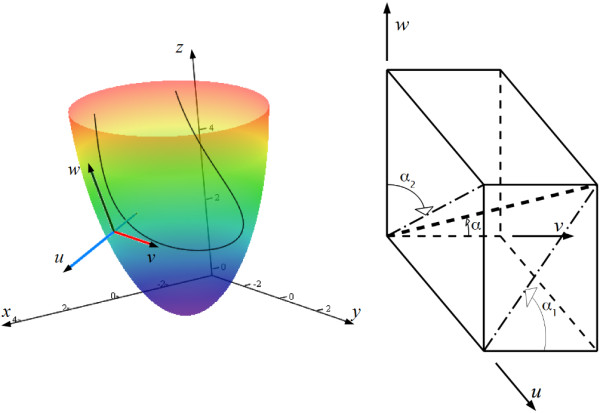
**On the left, definition of the local coordinate system.***Oxyz* is the global Cartesian coordinate system. The blue axis is normal *u* to the epicardium; the red axis, latitude *v*; the dark green axis, longitude *w*. The colourful surface is the epicardium; the colour depends on altitude *z*. The curve inside is a fibre as a chord image, the curve does not lie on the epicardium. The normal axis intersects the curve at a point. On the right, definition of the true fibre angle, *α*; the helix angle, *α*_1_; and the longitudinal angle, *α*_2_. The thick, dashed line is a tangent to a myofibre segment constructed at the origin of the coordinates. The dashed-and-dotted lines are projections of the myofibre tangent.

To represent fibre orientation and to compare it with anatomical data, Streeter [21] used the following angles: true fibre angle, *α*; the helix angle, *α*_1_; and the longitudinal angle, *α*_2_ (see Figure 9 for their definitions). We follow the same approach in this work.

Together with (22), (23) forms the basis for the LV model. Finally, we need to set proper fibre angles at the epicardial and endocardial surfaces.

**Figure 10:**
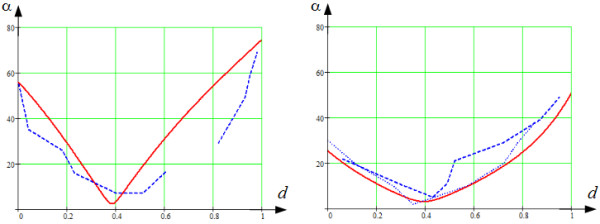
**The true fibre angle, *α *, (in degrees) in the equatorial and bottom areas of the LV.** The solid red lines correspond to the data from our model, the dashed and dotted blue ones correspond to the experimental data from [21]. The horizontal axis from endo- (value 0) to epicardium (value 1). The trabecular LV zone is not taken into account.

In the model given by (22) and (23), angle *α* depends on point position in the LV wall and changes from 90° on the endocardium to approximately 0° in the middle of the wall and then to 90° on the epicardium. In real hearts, the rotation of fibre is less, and its values at the endocardium and epicardium are about 60° and 70° ([21], Figure thirty-three). To account for that, we use only part of the interval 0≤*γ*_0_ ≤ *γ* ≤ *γ*_1_ ≤ 1. For example, if *γ*_0_ = 0.1 ≤ *γ* ≤ 0.75 = *γ*_1_, then the extreme angle values in the equatorial area are 55° at the endocardium and 75° at the epicardium (see Figure 10 and Results for more details).

As a change in the range of *γ* changes the anatomy of the heart, we need to rescale it to the normal heart using:

$$\begin{array}{l} R_{b}=R_{b}^{e}+\frac{L^{e}\gamma_{0}}{\gamma_{1}-\gamma_{0}},\\ L=\frac{L^{e}}{\gamma_{1}-\gamma_{0}},\\ Z_{b}=Z_{b}^{e}+\frac{h^{e}\gamma_{0}}{\gamma_{1}-\gamma_{0}},\\ h=\frac{h^{e}}{\gamma_{1}-\gamma_{0}},\\ \phi_{\text{max}}=\frac{\phi_{\text{max}}^{e}}{\gamma_{1}-\gamma_{0}}. \end{array}$$

After choosing of *γ*_0_ and *γ*_1_, we completely specify parameters of our model and can use it for generation of anisotropy.

## Comparison of the theoretical model with experimental data

In this section, we indicate the parameter values used in our study and compare theoretically obtained results with experimental data from [21] and [22].

### Verification of the model: comparison with Streeter’s data

We used the following parameter values reported in ([21], Table two): external radius of LV at the equator $R_{b}^{e}=33$ mm, thickness of LV wall at the equator *L*^*e*^ = 10 mm, height of under-equatorial LV part $Z_{b}^{e}=60$ mm, thickness of LV wall at the apex *h*^*e*^ = 7 mm, *ϵ*^*e*^ = 0.9; and we set angle of spiral surface torsion $\phi_{\text{max}}^{e}=3\pi$ according to ([21], Figure three c).

**Figure 11:**
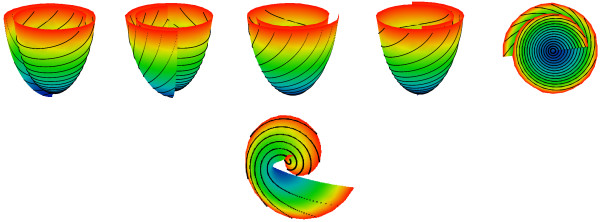
**ESS used in the model of convex LV with a thick equator and *γ * restricted to [*γ***_***0***_***,γ***_***1***_**] = [0.1,0.75]and chord images (black lines).** The first row: four side and one top views. The bottom figure: area of the apex (0 ≤ *z* ≤ *h*), top view.

See Figure 11 and 3 additional movie files for the spiral surfaces we made using these parameter values.

We used the parameters of the subepicardial and subendocardial boundaries *γ*_0_ = 0.1, and *γ*_1_ = 0.75, which gives *α* = 55° at the endocardium and *θ* = 75° at the epicardium that fits with ([21], Figure forty-four) taking into account the experimental measurement error and data variability.

We compared three angular characteristics of our myofibres field with experimental data from [21]. The comparison was made in two areas of the LV: the equatorial (58 mm ≤*z* ≤ 60 mm) and the bottom (18 mm ≤ *z* ≤ 21 mm) parts of the LV. In both regions, angles were compared along a line orthogonal to the epicardium, which is common in anatomical studies.

**Figure 12:**
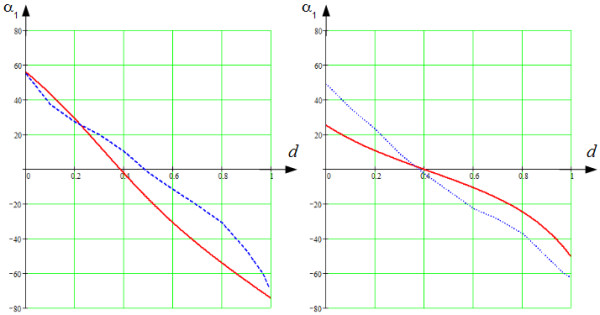
**The helix angle,*α***_***1***_**.** The axes are the same as in Figure 10.

**Figure 13:**
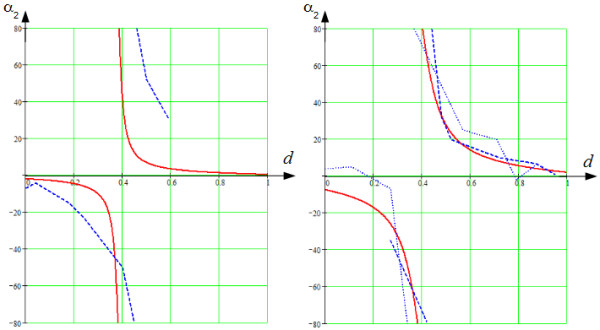
**The longitudinal angle,*α***_***2***_**.** The axes are the same as in Figure 10.

The results of comparison are shown in Figures 10, 12 and 13.

#### LV top zone

Note that in the LV top zone, the true fibre angle *α* varies non-monotonically, as in Streeter’s data [21]. In particular, *α* on the endocardium was close to 55°, on the epicardium it was close to 75° and decreased to 5° approximately at the middle of the LV wall, between 35% and 40% of the wall depth (Figure 10, on the left). We see some differences in the slope of curves in our model and in measured data; however, a qualitative correspondence is observed.

Figure 12, on the left, shows similar results for the helix angle. We see that going from endocardium to epicardium, the helix angle *α*_1_ decreased monotonically from +55° to -75° and was equal to 0° approximately at the middle of the LV wall, between 35% and 40% of the wall depth. We also see a good agreement of our model with the experimental results from [21].

As we see in Figure 13, on the left, the longitudinal angle, *α*_2_, going from endocardium to the middle of the LV wall, decreased from 0° to -90°. At the middle of the LV wall, between 35% and 40% of the wall depth, the experimental and modelled angle abruptly changed from -90 to +90°, which is an artefact. We found the jump at 43%, while it was 38% in Streeter’s data. At the exterior half of the LV wall, the *α*_2_ angle decreased at a decelerating rate to 0°. Here too, we observe good qualitative correspondence of our model with experimental data, although some quantitative differences in the slopes of the dependencies are present.

#### LV middle zone

The true fibre angle *α* also reaches a minimum in the mid-wall region, both in our model and in Streeter’s data. On the endocardium, it was close to 35°, on the epicardium, it was close to 50° and decreased to 5° approximately at the middle of the LV wall, at 0.4 of the wall depth (Figure 10, on the right).

Figure 12, on the right, displays similar results for the helix angle. We see that going from endocardium to epicardium, the helix angle, *α*_1_, decreased monotonically from +25° to -50° and was equal to 0° approximately at the middle of the LV wall, at 0.4 of the wall depth. We also see a good agreement of our model with the experimental results from [21].

In Figure 13, on the right, the longitudinal angle, *α*_2_, decreased from -5° to -90° between endocardium and mid-wall. At the middle of the LV wall, between 0.35 and 0.4 of the wall depth, the angle again abruptly changed to +90°. On the exterior half of the LV wall, the *α*_2_ angle decreased at a decelerating rate to 0°. A good qualitative correspondence with anatomical data is found in the outer two-thirds of the LV wall.

**Figure 14:**
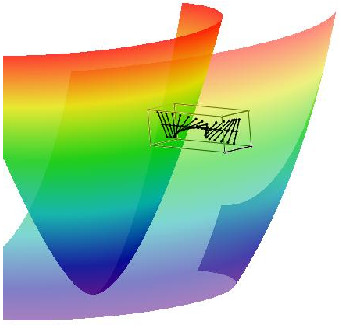
**Fibre slope angle variation depending on fibre’s position in the thickness of the wall in the LV model.** Endo- and epicardial wall surfaces are highlighted. Colour of the border surfaces from the equator to the vertex matches rainbow colours.

Our model also successfully reproduces the distribution of fibre directions in the thick, radially placed layer of the LV wall, as shown in Figure 14. Streeter studied the pattern and named it ‘Japanese fan’ ([21], Figure forty-two c).

Overall we can claim that our model adequately reproduces the direction of myocardial fibres in the human LV.

### Comparison with Auckland canine dataset

**Figure 15:**
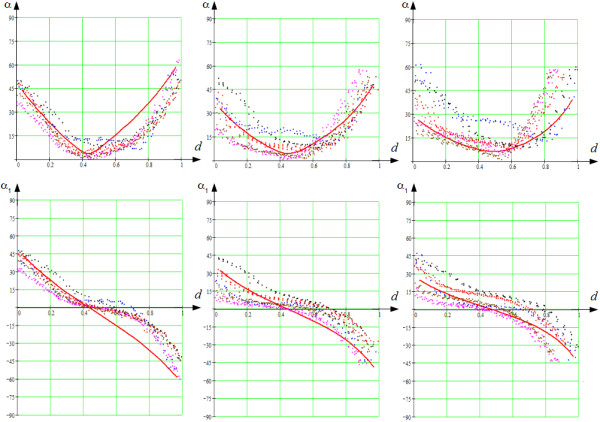
**The true fibre angle, *α *, (the first row) and the helix angle,*α***_***1***_**, (the second row) in the canine LV free wall.**vThe solid, red lines correspond to the data from our model, and the dots correspond to the Auckland canine anatomy data from [22]. The left graphs show the upper area of the LV (*ψ* = 30° at the epicardium); the centre graphs, the middle area (*ψ* = 45°); and the right graphs, the bottom area (*ψ* = 60°). The abscissa axis reflects the position of a point in the thick layer of the LV wall, 0 corresponds to the endocardium (the trabecular LV zone is not taken into account), 1 corresponds to the epicardium. The colour of the points corresponds to the five different longitudes (*ϕ*) at the LV free wall.

We also compared our model to the data used in [22]. The comparison was conducted in the following way. We aligned our LV model with the Auckland model along the vertical axis and found that reasonable fit of our anatomy to the Auckland heart model occurs for: $R_{b}^{e}=45$ mm, $Z_{b}^{e}=80$ mm, *h*^*e*^ = 12 mm, *L*^*e*^ = 15 mm, *ϵ*^*e*^ = 0.85, $\phi_{\text{max}}^{e}=3\pi$, *γ*_0_ = 0.15, *γ*_1_ = 0.9. Subsequently we constructed five meridional half-planes so that they divided the LV free wall region and the dihedral angle to four equal dihedral angles. We examined only points lying near the five half-planes. We used the same comparison procedure that we described in Section “Verification of the model: comparison with Streeter’s data”. The fibre orientation was compared at three different “latitudes” (close to apex, in the middle and close to base) along lines orthogonal to the heart surface. We computed the *x* values (they show the position of a point in the ventricular wall) and the two angles, *α* and *α*_1_, and plotted their values from the experimental dataset and from the theoretical model. The results obtained are shown in Figure 15.

We see that the plots of the *α* angle have characteristic V-shaped forms, both in the model and in four of five meridional sections at all three “latitudes”. The model angle is within the limits of the section angles in almost all the positions, except the external one-third of the wall on the left graph. The plots of the *α*_1_ angle show the same pattern: they descend from some positive values to some negative ones, speed of the decrease is higher in the endo- and epicardial area and slightly less in the mid-myocardium.

Note that we show only one line for our model. This is because our model is rotationally symmetric, and all five lines for different rotation angles are the same. The model is an idealization and it represents only some averaged characteristics of LV and neglects possible individual aspects of the LV form.

Our simulation programme is available on request.

## Discussion

In this section, we discuss limitations of our model, experimental methods and models that can be used to verify it, its possible applications and ways of improving it.

### Limitations

Our model adequately reproduces fibre angles at the LV middle zone, but agreement for the LV top and apex zones is mainly qualitative.

Inaccurate reproduction of fibre direction in the basal and apical zones can be caused by different reasons, one of which can be the idealized axisymmetrical LV form in the model. Real ventricles have essential deflections from the axis symmetry; they are somewhat individual, that is specific for every LV. In this work, we tried to construct an axisymmetrical model that could maximally suit the middle (by height) LV area. The apex and base of a real LV could considerably move away from the axis. We are currently working on extensions of the model which will adapt the idealized model to individual peculiarities of real ventricles. We hope to achieve more accurate imitation of the transmural run of the fibres at the apical and basal zones.

### Experimental methods that can be used to verify our model

Currently, there are several experimental methods that can be used to measure fibre orientation in the heart. One of them is the diffusion tensor imaging (DTI) technique. In this approach, a researcher finds the diffusion matrix of water molecules in the heart. The main directions of diffusion are determined by the structure of the tissue [24-27], and by calculating the eigenvectors of the matrix corresponding to the largest eigenvalue, the fibre direction can be found. Diffusion tensor magnetic resonance imaging (DT-MRI) measurements can be done with spatial resolutions up to 200 *μ*m [28]. Another advanced technique is the micro computerized tomography (Micro-CT) imaging. Micro-CT measurements can be done with spatial resolutions up to 36–70 *μ*m [29], and both these methods produce high-quality data that can be used in computer models.

Direct measurement of anisotropy also can be conducted via tedious histological studies of fibre direction in 3-D [21,22]. In this method, the researcher makes a series of parallel sections of the heart. In each section, angles of fibre slope are measured, which gives a full picture of the fibre directions in the heart. Recently, Smaill et al. developed a combined high-resolution serial imaging microscopy technique [30]. In this method, after heart fixation, they perform cross-section and make successive, high-resolution images of the heart. Then, using computer processing, the data are collected to form an overall 3-D dataset.

In [31], the myofibres in the foetal human heart are investigated using quantitative polarized light microscopy. The hearts are embedded in a transparent resin, polymerized and then sectioned. Afterward, the elevation and azimuth angles are measured by means of polarized light (see [31] for details).

### Comparison with other models

Experimental data on the fibre orientation obtained as described above may be in different ways used for construction of anatomical computational models: 

• either as a discrete dataset in finite element models [32-34];

• or for the verification of rule-based models, i.e. the models formed on the base of some constitutive rule [35-38].

One of the most recent rule-based methods is a Laplace–Dirichlet algorithm [37], which takes a noisy DTI-derived fibre orientation field as input data and yields, firstly, the transmural and apicobasal directions for the entire myocardium and, secondly, a smooth and continuous fibre orientation field. Another approach was used by Peskin, who derived fibre orientation field from the principles of mechanical equilibrium [39]. One more anatomic model based on a principle of the mechanical activity of the heart was a model by Chadwick, who considered a cylindrical LV and specified the helix angle linearly depending on point position in the LV wall [40]. Beyar et al. shaped LV into a spheroid and also used linear dependence of the helix angle on the distance between any point in the ventricle wall and the endocardium [2]. An interesting example of the theoretical approach was developed by Arts et al. in 1992 [41]. They constructed a model of an ellipsoidal LV, complicated the law of helix angle change to a piecewise quadratic one and quantified orientation of the muscle fibres via the helix fibre angle distribution, which was found upon application of the mechanical adaptative principle suggested by Arts et al. in 1982 [42].

In this work we present our rule-based model focused on the LV morphology including simulation of the ventricle shape and fibre orientation in its wall. The developed formalism is substantially associated with both ventricle band concept of cardiac architecture given by Torrent-Guasp [13] and anatomic observations presented by Streeter in his classical work [21]. In our approach to the modelling of the LV architecture, anisotropy of the heart was derived from some general principles. In our model, the LV is considered a set of identical spiral surfaces combined with each other by rotation about the vertical axis. Every spiral surface is defined analytically and represents a mapping of a half disc. The first step of the transformation is the mapping of the semicircle to a conical surface. In the second step, the conical surface is transformed to a curved spiral surface, representing the quasi-elliptical shape of the LV boundary surface. Finally, every spiral surface is filled with myocardial fibres, represented by the transformed images of the chords that were parallel to the diameter in the initial semicircle (see Figure 1).

Our model is not the only wrapping-based myocardium model. Sinha et al. proposed in [43] a model of one myocardial layer which had a rectangular form and was wrapped around a (truncated) cone. They used this very simple model to study termination of re-entrant waves rotating around obstacles in cases of isotropy and anisotropy but without any linkage to the real fibre pattern in the heart ventricles.

We used experimental data from the above cited work by Streeter [21], as well as from other more recent works [22] for the model verification. In particular, the model proved to reproduce adequately both the looping arrangement of the muscle fibres and the specific 3-D pattern of the relative positions of the fibres in the transmural direction through the ventricle wall.

These accurate reproductions allow us to consider the model a touchstone in validating the ventricle band concept of cardiac architecture originated by Torrent-Guasp, because the model, based on this concept, yields an adequate fibre field as a consequence of the postulates.

It seems reasonable to compare our model with another rule-based model that assigns fibre orientation locally, particularly with the very interesting and promising model by Bayer et al., mentioned earlier in this section [37]. For this comparison, only the reproduced fibre orientation in various parts of the LV of the two models can be used. The Bayer model is based on DT-MRI data of anisotropy in two ventricles of a canine heart. The average angle divergence between the model and the DT-MRI fibre directions is 23°; that is there is not a complete quantitative matching of the real experimental and reproduced data, but there is reasonable concordance. Specifically, Bayer’s model quantitatively reproduces fibre anisotropy in the basal and apical LV zones better than our model (see [37], Figure three). In the section describing the limitations of our model, we point to this quantitative inaccuracy in our model and propose some ways to eliminate it. At the same time, our model better reproduces the experimental data in the middle LV zone; namely, we obtain the specific s-like plot of the angle *α*_1_ in the transmural direction (see Figure 15, the bottom right panel). In Bayer’s model, this dependency is linear by definition. Moreover, if we follow the cited paper by Bayer et al. ([37], Eq. (1), (2)), all plots for angle *α*_1_ presented in Figure 12 reveal independence of the angle from both the latitude and the longitude of the intramural position within the wall. Bayer and co-authors suggest that their model can be easily improved to take the non-linearity of angle *α*_1_ into account. But, it also is necessary to make the anisotropy latitudinally and longitudinally independent, and it is not easy to do so. Our model reproduces such a dependence (see Figures 10, 12, 13, 14 and 15), which is proper for real hearts, and does it quite fairly for the middle zone of the LV.

One more simplification of Bayer’s model concerns transmural rotation of the fibres’ directions, named ‘Japanese fan’ by Streeter ([21], Figure forty-two c). In that model, the rotation is defined in one plane only, that is, around only one axis, settled transmurally. This plane lies tangentially to the surface determined by the condition *d* = const, where *d* is a term specified in the cited paper by Bayer et al. [37] and presents there the occurrence depth of particular locus within the wall; for example, *d* = 0 on the endocardium and *d* = 1 on the epicardium. Moreover, if we assess results obtained in Bayer’s model by means of the angle *α*_3_ defined by Streeter [21] and determine transmural direction of the fibre orientation, it will prove to be constantly 0, which is a substantial simplification. This feature does not allow mapping the 3-D pattern of the relative positions of the fibres in the transmural direction through the ventricular wall.

In contrast, in our model the 3-D pattern is taken into consideration (see Figures 10, 12, 13, 14 and 15, and note that in the middle LV zone these angles are reproduced quite well).

Thus, we believe that both models have their own virtues and their own limitations, and further development of the models would be useful to overcome the limitations.

### Development and uses of our model

We suggest that analytical representation of the geometry presented here can be used for development of new numerical methods to study electrical and mechanical activity of the heart. As our model provides analytical mapping of a rectangle in (*γ*,*ψ*,*ϕ*) space into the curvilinear heart shape, one can formulate a rectangular numerical scheme in (*γ*,*ψ*,*ϕ*) space (where representation of boundary conditions is simplest) and account for anisotropy by explicit analytical formulae. The model can also be used to generate various anisotropic properties of the heart and modulations of the LV shape (via model parameter variations) and to study their effects on electrical and mechanical heart functions.

Of course, our model is an idealization; it represents some averaged characteristics of the LV and neglects possible individual aspects of the LV form. Model adaptation to individual characteristics is the subject of a particular line of research. We are developing methods to further modifying the LV model to customize it to individual hearts.

## Conclusions

We have constructed one of the simplest analytical descriptions of cardiac anatomy based on the Torrent-Guasp’s ventricular band concept. The model can be used for verification of the band concept as well as for various numerical simulations to study the effects of anisotropy on cardiac excitation and mechanical function. Our model shows good qualitative agreement between the simulated fibre orientation field and the experimental data on LV anisotropy.

## Appendix

### Mapping a point on the semicircle to the spiral surface

In this section, we give all needed formulae to map a point on the semicircle to the cone and then to the spiral surface. The following input parameters are used 

• external radius of LV at the equator, *R*_*b*_;

• thickness of LV wall at the equator, *L*;

• height of LV, *Z*_*b*_;

• thickness of LV wall at the apex, *h*;

• conicity-ellipticity parameter, *ϵ*;

• angle of spiral surface torsion, *ϕ*_max_.

Let us consider a point (P,*Φ*) on a semicircle with a radius *K*. In order to get outer radius *R*_*b*_ of the LV on the equator, we need to use

$$K=\frac{\phi_{\text{max}}}{\pi }\cdot R_{b}.$$

The image of the point on the ESS has the following coordinates (see also (23)):

$$\begin{array}{lll} \rho (P,\Phi ) & =P\cdot \frac{\pi }{\phi_{\text{max}}}\cdot \left(1-\frac{\Phi }{\pi }\cdot \frac{L}{R_{b}}\right),\; & \\ \phi (P,\Phi ) & =\phi_{\text{max}}\cdot \frac{\Phi }{\pi }.\; & \end{array}$$

The mapping (*x*,*y*,*z*) → (*γ*,*ψ*,*ϕ*) is given by

$$\begin{array}{lll} & \rho =\sqrt{x^{2}+y^{2}},\; & \\ & \gamma :\;z_{\epsilon \text{sp}}(\rho ,\gamma )=z,\; & \\ & \gamma^{\prime }=1-\gamma ,\; & \\ & \psi =\text{arcsin}\left(\frac{Z_{s}-z}{Z_{b}-\mathrm{\gamma \prime h}}\right),\; & \\ & \phi =\text{a}\text{tan}2(y,x).\; & \end{array}$$

The fibre direction $\vec{v}=(\text{vx},\text{vy},\text{vz})$ at a point (*γ*,*ψ*,*ϕ*):

$$\begin{array}{lll} \rho & =\rho_{\text{mid}}(\psi ,\gamma^{\prime }),\; & \\ \rho_{\text{pl}} & =\frac{\rho \phi_{\text{max}}}{\pi (1-\mathrm{L\gamma \prime}/R_{b})},\; & \\ \phi_{\text{pl}} & =\pi \gamma^{\prime },\; & \\ t & =\frac{\rho }{R_{b}-\gamma^{\prime }L},\; & \\ Y & =\rho_{\text{pl}}\text{sin}\phi_{\text{pl}}\;\;\text{(see Figure 1),}\; & \\ \frac{\mathrm{d\rho}}{d\phi_{\text{pl}}} & =-\frac{\rho_{\text{pl}}}{R_{b}\phi_{\text{max}}}\cdot \left(L+\frac{\pi R_{b}-\phi_{\text{pl}}L}{\tan\phi_{\text{pl}}}\right),\; & \\ \frac{\mathrm{d\phi}}{d\phi_{\text{pl}}} & =\phi_{\text{max}}/\pi ,\; & \\ F & =F_{\epsilon \text{sp}}(t)\;\;\text{(see(22)),}\; & \\ \mathrm{F\prime} & =F_{\epsilon \text{sp}}^{\prime }(t)=\frac{1-\epsilon -\epsilon (1-\epsilon -t)/\sqrt{\epsilon^{2}+2t(1-\epsilon )-t^{2}}}{{\left(\epsilon -1\right)}^{2}+\epsilon^{2}},\; & \\ \frac{\text{dz}}{\mathrm{d\rho}} & =\frac{Z_{b}-\gamma^{\prime }h}{R_{b}-\gamma^{\prime }L}\cdot F^{\prime },\; & \\ \frac{\text{dz}}{\mathrm{d\phi}} & =\frac{1}{\phi_{\text{max}}}\cdot \left(h(1-F)+\frac{F^{\prime }\mathrm{\rho L}(Z_{b}-\gamma^{\prime }h)}{{\left(R_{b}-\gamma^{\prime }L\right)}^{2}}\right),\; & \\ \text{vx} & =\frac{\mathrm{d\rho}}{d\phi_{\text{pl}}}\text{cos}\phi -\rho \text{sin}\phi \frac{\mathrm{d\phi}}{d\phi_{\text{pl}}},\; & \\ \text{vy} & =-\left(\frac{\mathrm{d\rho}}{d\phi_{\text{pl}}}\text{sin}\phi +\rho \text{cos}\phi \frac{\mathrm{d\phi}}{d\phi_{\text{pl}}}\right),\; & \\ \text{vz} & =\frac{\text{dz}}{\mathrm{d\rho}}\cdot \frac{\mathrm{d\rho}}{d\phi_{\text{pl}}}+\frac{\text{dz}}{\mathrm{d\phi}}\cdot \frac{\mathrm{d\phi}}{d\phi_{\text{pl}}}.\; & \end{array}$$

## Abbreviations

LV: Left ventricle; DT(I): Diffusion tensor (imaging); MRI: Magnetic resonance imaging; CT: Computer tomography; GF: Generating function.

## Competing interests

The authors declare that they have no competing interests.

## Authors’ contributions

SFP did the mathematical and programming work and wrote a draft of the main part of the article. VIB took part in the mathematical conception formulation and discussion of the results. AVP participated at a later stage of the research on model verification, proposed the idea on epi- and endocardial anisotropy fit and took part in writing the paper. LBK formulated the basic ideas of the mathematical construction, took part in writing Abstract, Introduction, Discussion, and Conclusions, editing the paper and discussion of the results. OES took part in discussion of the mathematical construction, proposed the ideas for numerical algorithms, experiments and visualisation, took part in discussion of the results and editing the paper. VSM took part in the physiological conception formulation, comparison of the results with the real morphological data and discussion of the model validity. All authors read and approved the final manuscript.

## Supplementary Material

Additional file 1 **Rotating plane curve in space.** On the left, changing curve on plane; on the right, the same curve moves and rotates in space. Click here for file

Additional file 2 **Traced rotating plane curve in space.** The same movement of the same curve, but with its trace, which is a spiral surface. Colour is linked with height *z*, red codes 0, purple codes 60 mm. Click here for file

Additional file 3 **Lower part of the spiral surface.** It is the lower part (0 ≤ *z* ≤ 15 mm) of the forming spiral surface from Additional file 2. Here we see the apical zone clearer. Colour is linked with height *z*, red codes 0, purple codes 15 mm. Click here for file
